# Expression of the Glioma-Associated Oncogene Homolog 1 (Gli1) in Advanced Serous Ovarian Cancer Is Associated with Unfavorable Overall Survival

**DOI:** 10.1371/journal.pone.0060145

**Published:** 2013-03-28

**Authors:** Alessandra Ciucci, Ilaria De Stefano, Valerio Gaetano Vellone, Lucia Lisi, Carolina Bottoni, Giovanni Scambia, Gian Franco Zannoni, Daniela Gallo

**Affiliations:** 1 Department of Obstetrics and Gynecology, Catholic University of the Sacred Heart, Rome, Italy; 2 Department of Histopathology, Catholic University of the Sacred Heart, Rome, Italy; 3 Institute of Pharmacology, Catholic University of the Sacred Heart, Rome, Italy; Dresden University of Technology, Germany

## Abstract

Recent evidence links aberrant activation of Hedgehog (Hh) signaling with the pathogenesis of several cancers including medulloblastoma, glioblastoma, melanoma as well as pancreas, colorectal, and prostate carcinomas. Here we investigated the role of the transcription factor Gli1 in ovarian cancer. To this end, the expression profile of Gli1 was examined in normal ovaries, ovarian tumors, and ovarian cancer cell lines, and the *in vitro* effects of a specific Hh-pathway blocker, KAAD-cyclopamine, or a specific Gli1 inhibitor (GANT58) on cell proliferation and on Hh target gene expression were also assessed. Results obtained showed that epithelial cells in ovarian cancer tissue express significantly higher levels of nuclear Gli1 than in normal ovarian tissue, where the protein was almost undetectable. In addition, multivariate analysis showed that nuclear Gli1 was independently associated to poor survival in advanced serous ovarian cancer patients (HR = 2.2, 95%CI 1.0–5.1, p = 0.04). *In vitro* experiments demonstrated Gli1 expression in the three ovarian carcinoma cell lines tested, A2780, SKOV-3 and OVCAR-3. Remarkably, although KAAD-cyclopamine led to decreased cell proliferation, this treatment did not inhibit hedgehog target gene expression in any of the three ovarian cancer cell lines, suggesting that the inhibition of cell proliferation was a nonspecific or toxic effect. In line with these data, no differences on cell proliferation were observed when cell lines were treated with GANT58. Overall, our clinical data support the role of Gli1 as a prognostic marker in advanced serous ovarian cancer and as a possible therapeutic target in this disease. However, our *in vitro* findings draw attention to the need for selection of appropriate experimental models that accurately represent human tumor for testing future therapies involving Hh pathway inhibitors.

## Introduction

Epithelial ovarian cancer is one of the leading causes of death in female malignancies in the great majority of developed countries [1]. Indeed, since this disease has few symptoms at earlier stages of its development, the majority of women are diagnosed after the primary tumor has already metastasized; despite the initial response to surgical debulking and the first-line therapy with carboplatin and paclitaxel, most tumors eventually develop drug resistance and the 5-year survival is generally below 30% [2]. Although many efforts have been made to clarify the etiology of ovarian carcinogenesis and the molecular mechanisms involved in proliferation of ovarian carcinoma cells, this disease remains among the less understood of major human malignancies.

The Sonic hedgehog (Shh) signal-transduction pathway is important in regulating patterning, proliferation, survival and growth of many cells and tissues, in the embryo and the adult. Binding of the secretory Hh ligands (Sonic, Indian, or Desert, SHH, IHH or DHH) to their transmembrane receptor Patched (Ptch1) initiates the classical Hh signaling pathway by releasing Smoothened (Smo) from Ptch1-dependent suppression. Smo modulates a cytoplasmic complex containing Suppressor of Fused (Sufu), thus activating the 3 glioma-associated (Gli) transcriptional regulators. Gli1 induces and Gli3 represses Hh target genes that include *Gli1*, *PTCH1*, *Cyclin D1*, *c-Myc*, and *Bcl-2*, whereas Gli2 can act in either a positive or negative manner depending on posttranscriptional and posttranslational processing events [3].

Aberrant activation of Hh signaling has been implicated in several cancers, including skin, brain, colon, lungs, prostate, blood and pancreas among others, driven either by a ligand dependent or a ligand-independent pathway [4], [5]. Ligand dependent Hh pathway may either be autocrine, where the Hh ligand produced by tumor cells act on neighboring tumor cells causing cell-autonomous pathway activation, or may involve a paracrine mechanism where Hh ligand secreted from the epithelium signals the underlying stromal compartment to create a favorable microenvironment that supports tumor growth. Autocrine signaling is seen in case of lung and prostate cancer, whereas in case of ovarian and colorectal cancer the paracrine mechanism seems prevalent [4], [5]. In the ligand-independent signaling, the Hh pathway is activated in a cell-intrinsic manner through loss-of-function mutations in negative-acting components, such as Ptch1 and Sufu, or through gain-of-function mutations in positive-acting components, such as Smo [4]. Besides, the Hh–Gli signaling interacts with other signaling pathways in bidirectional and context-specific ways, and several lines of evidence suggest that the context-dependent regulation of the Gli code by oncogenes and tumor suppressors constitutes a basis for the widespread involvement of Gli1 in human cancers, this protein supporting tumor progression and metastatic transition [6], [7]. Remarkably, in ovarian cancer, the Hh pathway might also interact with the estrogen signaling in a complex network modulating the phenotypic plasticity [8].

In the present study we sought to determine whether the Hh pathway is activated in ovarian cancer. To this end we first investigated immunohistochemically the expression of Gli1 protein in normal ovarian surface epithelium and in advanced serous ovarian cancers and correlated its expression with clinicopathological parameters and patient outcomes. Next, we examined the expression of Gli1 in ovarian cancer cell lines, and the *in vitro* effects of a specific hedghehog pathway blocker, KAAD-cyclopamine [9], [10] or a specific Gli1 inhibitor, GANT58 [11], on cell proliferation and on hedgehog target gene expression. Gli1 is actually considered as the ultimate and thus crucial transcriptional activator of the Hh pathway; its transcription is induced by Hh signaling, making it, so far, the best dependable marker of an active pathway, consistently transcribed in Hh-responding cells [7]. Besides, Gli1 has been considered a suitable target for cancer therapy because it is downstream of several signaling pathways [12].

## Materials and Methods

### Ethics Statement

This study obtained approval from the Ethical Committee of the Catholic University of Sacred Heart, Rome, Italy and patients gave written consent for tissue collection and analyses.

### Patients

The study included 56 patients with advanced serous ovarian cancer admitted to the Gynecologic Oncology Unit, Catholic University of Rome, between March 2000 and December 2008. Macroscopically and histopathologically normal ovaries, removed for prophylactic reasons, were also obtained from 12 postmenopausal women. Clinicopathological characteristics of the overall series are summarized in Table 1. According to standard guidelines, maximal surgical effort was attempted in all patients resulting in complete resection (residual tumor 0 mm) in 35 (63%) cases. All patients received platinum-based chemotherapy (75–100 mg/m^2^ for cisplatin, AUC = 5 for carboplatin, per cycle). Fifty patients (89.3%) also received paclitaxel (135–175 mg/m^2^ for each cycle). Recurrence of disease was defined according to GCIG CA125 criteria [13], [14] and/or radiological confirmation of tumor progression. To define chemosensitivity, we used the common definition of platinum resistance, defining as “sensitive” patients that relapsed 6 months or more after prior platinum-containing chemotherapy, and as “resistant” patients that relapsed less than 6 months after chemotherapy was stopped, or that progressed while on therapy [15]. Follow-up data were available for all 56 patients (median follow-up, 35 months; range, 9–127 months). During the follow-up period, progression and death of disease were observed in 42 and 23 patients, respectively.

**Table 1:** Clinicopathological features of the overall series.

Characteristics	No. of patients (%)
**All cases**	56
**Median Age (range)**	54 (33–79)
**Grade**	
G1	2 (3.6)
G2	10 (17.8)
G3	44 (78.6)
**FIGO Stage**	
III	54 (96.4)
IV	2 (3.6)
**Residual tumor**	
0 mm	35 (62.5)
>0 mm	21 (37.5)
**Primary chemotherapy**	
Platinum/paclitaxel	50 (89.3)
Platinum-based	6 (10.7)
**Chemosensitivity**	
Sensitive	40 (71.4)
Resistant	16 (28.6)

### Immunohistochemistry

Immunohistochemistry was performed as previously described [16]. Briefly, antigen retrieval procedure was performed by microwave oven heating in citrate buffer (pH = 6). Sections were incubated with 20% normal goat serum for 30 min at room temperature to reduce nonspecific binding. Cells expressing Gli1 (clone H-300, sc-20687, Santa Cruz Biotechnology, Santa Cruz, CA, dilution 1∶50), were identified after overnight incubation at 4°C. Antibody used in the present study was tested for specificity as described previously [17]–[20]. Sections were incubated with the secondary antibody (goat anti-rabbit) for 30 min, at room temperature. The slides were developed with diaminobenzidine (DAB substrate System, DakoCytomation), counterstained with Mayer’s Haematoxylin, dehydrated in ethanol and xylene, and finally mounted. Basal cell carcinoma was used as a positive control. Staining without primary antibody was used as a negative control.

### Evaluation of Immunohistochemical Staining

Expression was evaluated by considering the percentage of cells exhibiting immunoreaction in a minimum of 500 histologically identified neoplastic cells. Gli1 staining was observed both in the cytoplasm and in the nucleus in ovarian carcinoma cells. However, as Gli1 is a transcription factor, only nuclear Gli1 expression was scored for subsequent data analyses and correlation studies. Immunostaining was evaluated by two independent observers (GFZ and VGV) who were unaware of the patients’ diagnosis. To define a cut-off value for Gli1, we compared nuclear immunoreactivity measured in normal versus tumor tissues. Immunoreactivity was absent in epithelial cells of all but one normal ovaries, with a single specimen showing a weak staining reaction in about 10% of cells. Tumor sections in which >10% of cells were stained were thus consider to be positive.

### Cell Culture

The ovarian carcinoma cell lines A2780, SKOV-3 and OVCAR-3 were purchased from the European Collection of Cell Cultures (ECACC, Salisbury, UK). OVCAR-3 and A2780 cells were cultured in RPMI 1640 medium (Lonza, Basel, Switzerland), SKOV-3 were grown in Dulbecco’s modified Eagle’s medium (Lonza). The medium was supplemented with 10% fetal bovine serum (FBS, Lonza), 2 mM glutamine and antibiotics (100 mg/ml streptomycin and 100 IU/ml penicillin) (Lonza). All cultures were maintained at 37°C under a humidified atmosphere of 5% CO2 and 95% air.

### Immunocytochemistry

Cells (2.0 to 4.0×10^4^ cells/well) were plated into two-well chamber slide (Nunc® Lab-Tek® II Chamber Slide, Nunc, Inc., Naperville, IL). The cells were cultured for 24 h and used for immunostaining. Slides were washed twice with PBS, fixed with 4% paraformaldehyde and permeabilized with 0.5% Triton X-100. The endogenous peroxidase was blocked with 3% H_2_O_2_ for 5 min. After washing twice with PBS, cells were incubated with a blocking solution containing 20% normal horse serum in PBS for 30 min at room temperature. Excess blocking solution was drained, and samples were incubated with a 1∶50 dilution of anti-Gli1 antibody (clone H-300, sc-20687, Santa Cruz Biotechnology) overnight at 4°C in a humidified chamber. The samples were then rinsed three times with PBS and incubated with secondary antibody, EnVision System-HRP (Dako, Carpinteria, CA), for 30 min at room temperature. The immunoreactivity was detected using the 3,3′-diaminobenzidine substrate (DAB substrate System, Dako). The slides were counterstained with Mayer’s Haematoxylin, dehydrated in ethanol and xylene, and finally mounted.

### Proliferation Assay

A2780 (3.0×10^4^ per well), OVCAR-3 (2.0×10^4^ per well) and SKOV-3 (2.0×10^4^ per well) cells were seeded in 24-well plates in complete culture medium. After overnight incubation, the medium was changed to 2% FBS containing various concentrations of KAAD-cyclopamine (Santa Cruz Biotechnology), or GANT58 (Calbiochem, San Diego, CA). Substances were dissolved in absolute DMSO and diluted in the appropriate culture medium immediately before use. Control cells received the same amount of DMSO diluent. After 48 h of incubation, cells were harvested by trypsinisation, and viable cells were counted using Nucleocounter (Chemometec, Allerod, Denmark). All experiments were performed at least three times in duplicate.

### MTT Assay

All ovarian cancer cell lines were seeded (4.0×10^3^ per well) in 96-well plates in complete culture medium. After overnight incubation, the medium was changed to 2% FBS containing various concentrations of KAAD-cyclopamine (Santa Cruz Biotechnology) or GANT58 (Calbiochem). Cytotoxicity was quantified by measurement of the reduction of MTT (3-(4,5-Dimethylthiazol-2-yl)-2,5- diphenyltetrazolium bromide, a tetrazole) to produce a dark blue formazan product. MTT was added to each well 48 h after the beginning of the insult. After 3 h incubation, the Solubilization/Stop Solution was added to the culture wells to solubilize the formazan product, and the absorbance at 570 nm recorded using a 96-well plate reader (Victor 1420, Wallac, Perkin Elmer). All experiments were performed at least three times in duplicate.

### Western Blot Analysis

Whole-cell extracts were prepared after lysis in buffer containing 50 mM Tris-HCl, pH 7.4, 0.2 M NaCl, 1 mM EDTA, 1 mM EGTA, 1% Triton X-100, 0.5% NP-40, 10% Glycerol supplemented with phosphate and protease inhibitors. Equal amounts of protein (50 µg/sample) were separated by SDS gradient polyacrylamide gel electrophoresis (4–20%) (Bio-Rad Laboratories, Hercules, CA), transferred onto PVDF membranes (Immobilon-P transfer membrane, Millipore, Billerica, MA). The membranes were blocked for 1 hour with 5% (w/v) nonfat dry milk in Tris Buffered Saline containing 0.1% Tween 20 (TBST) and incubated with primary antibodies (Gli1: H300, Santa Cruz Biotechnology; β-actin: A5441, Sigma-Aldrich, St. Louis, MO) in TBST with 3% non-fat dry milk, at 4°C overnight. After washing three times with TBST, the membranes were labeled with horseradish peroxidase-conjugated secondary antibodies for 1 hour at room temperature. Specific proteins were visualized with the ECL Western blotting system according to the manufacturer’s instructions (Pierce Biotechnology, Rockford, IL). β-actin Western blot analysis was performed as a control of equal sample loading.

### mRNA Analysis in Real Time PCR

Total RNA was extracted using Total RNA isolation NucleoSpin RNA II (Macherey-Nagel GmbH & Co. KG) and was reverse-transcribed using RETROscript kit (Ambion®, Life Technologies Invitrogen, Carlsbad, CA), according to the manufacturer’s protocol. To evaluate quantitative changes in Gli1 mRNA levels, each RT reaction mixture was then subjected to real time PCR (Q-PCR) using the following cycling conditions: 35 cycles of denaturation at 95°C for 20 s; annealing and extension at 60°C for 20 s; using the Brilliant III Ultra-Fast SYBR® Green QPCR Master Mix (Stratagene, La Jolla, CA, USA). PCR reactions were carried out in a 20 µL reaction volume in a MX3000P real time PCR machine (Stratagene). The PCR primers for detecting specific transcripts were as follows: Gli1, 5′-GCCAGCCAAGAGAGACCAACAG-3′ and 5′-CCGACAGAGGTGAGATGGACAG-3′; β-actin 5′-ACG TTG CTA TCC AGG CTG TCC AT-3′ and 5′-TTA ATG TCA CGC ACG ATT TCC CGC-3′. Relative mRNA concentrations were calculated from the take-off point of reactions (threshold cycle, Ct) using the comparative quantitation method performed by Stratagene software and based upon the −ΔΔCt method. This analysis approximates a given sample’s target mRNA (Gli1) level relative to the mean of the target mRNA levels in untreated controls (“calibrator” value), thus permitting statistical analysis of deviation from the mean even among the controls. Ct values for β-actin expression served as a normalizing signal. In each assay, the PCR efficiency was also calculated using serial dilution of one experimental sample; efficiency values between 80 and 100% were found for each primer set and taken into account for the comparative quantitation analysis.

### Statistical Analysis

The expression of Gli1 and its association to clinicopathological parameters was evaluated using the Mann–Whitney nonparametric test. Disease-free survival and overall survival were calculated from the date of diagnosis to the date of progression/death, or the date last seen. The prognostic effect of the various parameters on clinical outcome (i.e. recurrence or death of disease) was tested by plotting survival curves according to Kaplan–Meier method, and comparing groups using the log rank test, as well as by multivariate analysis using the Cox model. Kaplan–Meier survival estimates were generated from date of histological diagnosis to time of the last follow up or death. In univariate analysis, each parameter was categorized for subsequent statistical analysis. Only variables with p-value ≤0.2 in the univariate analysis were included in multivariate model. Results obtained in *in vitro* experiments were expressed as mean ± SD and were analyzed by two-tailed Student’s t-test. Differences were considered significant if P≤0.05. All statistical analyses were performed using the GraphPad Prism5 Software (San Diego, CA, USA). Cox analysis was performed using the StatPlus 2009.

## Results

### Gli1 Expression in Advanced Serous Ovarian Cancers

**Figure 1:**
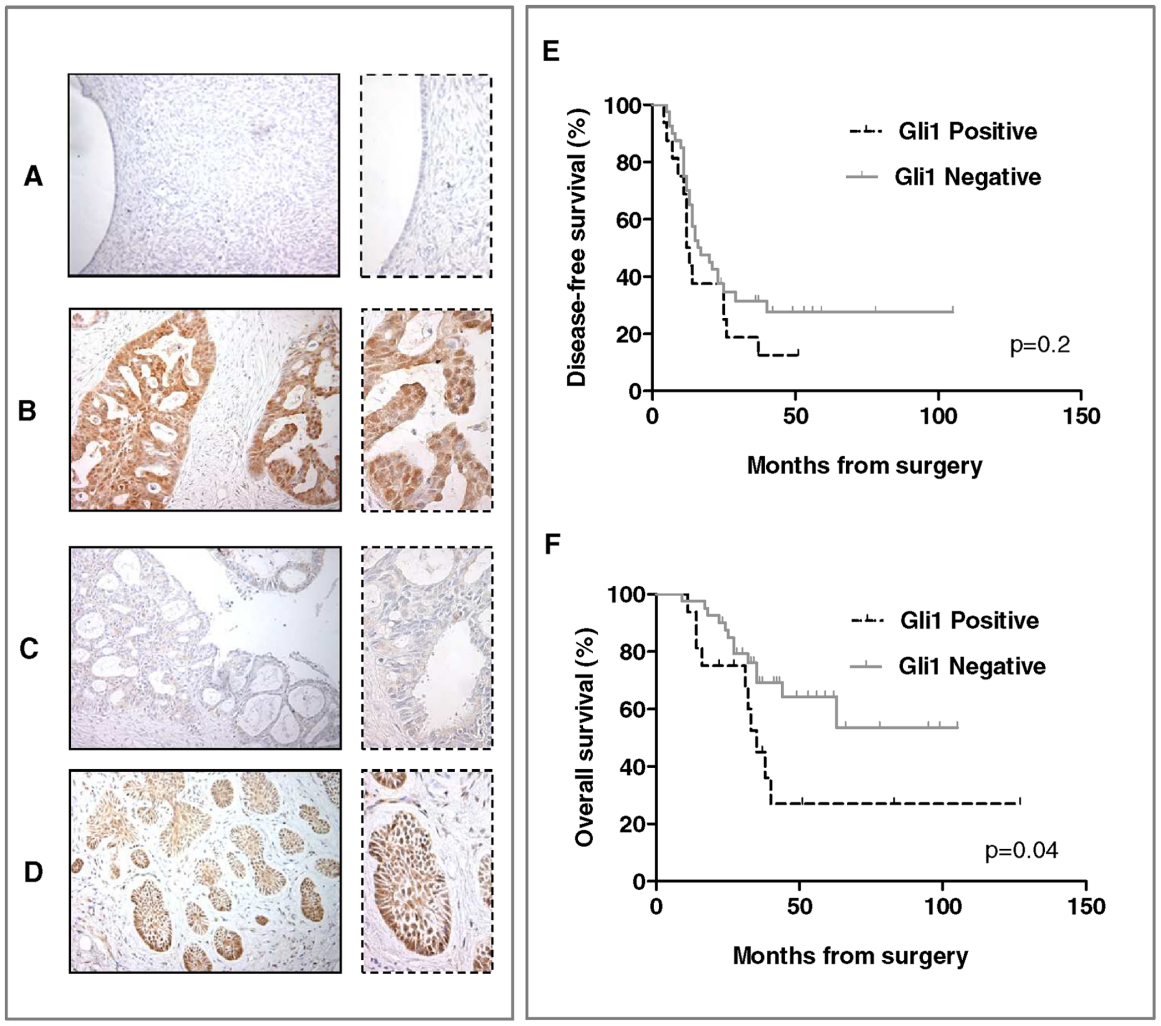
Gli1 expression in advanced serous ovarian cancer. Representative pictures for Gli1 immunostaining showing expression in normal ovary epithelium (**A**), and high (**B**) or low (**C**) expression in ovarian cancer tissues. Basal cell carcinoma was used as positive control (**D**). Magnification 20x, and 40x. (**E)** Kaplan–Meier survival curve for the probability of disease-free survival and (**F**) overall survival according to the expression of nuclear Gli1 in advanced serous ovarian cancer patients. Positive (>10%) Gli1 expression in the nucleus is significantly associated with overall survival disadvantage (p = 0.04).

A total of 56 primary advanced serous ovarian cancers and 12 normal ovarian tissues from postmenopausal women were evaluated. Gli1 immunoreactivity was absent in the surface epithelium, as well as in stromal cells, of the normal ovaries (Fig. 1A) in all but one case, this last showing a weak nuclear staining in about 10% of cells. A positive nuclear reaction was observed in 29% of the serous carcinomas (Fig. 1B); cytoplasmic immunoreactivity was also observed in the majority of specimens examined. These findings indicate that the Hh signal pathway is activated in ovarian carcinoma cells compared with normal epithelial cells.

Table 2 shows median nuclear immunoreactivity of Gli1 in advanced serous ovarian cancer patients according to clinicopathological parameters. No correlation was demonstrated between nuclear Gli1 expression and any of the parameters examined.

**Table 2:** Gli1 expression in the overall series.

Characteristics	No. of patients (%)	Percentage of Gli1-positive cells	
		Median (range)	p value
**All cases**	56		
**Age**			
≤54	30 (53.6)	0 (0–70)	0.1
>54	26 (46.4)	10 (0–60)	
**Grade**			
G1–2	12 (21.4)	5 (0–60)	
G3	44 (78.6)	5 (0–70)	0.6
**Residual tumor**			
0 mm	35 (62.5)	10 (0–70)	
>0 mm	21 (37.5)	0 (0–30)	0.7
**Chemosensitivity**			
Sensitive	40 (71.4)	5 (0–70)	
Resistant	16 (28.6)	5 (0–30)	0.9

The prognostic role of Gli1 expression for both DFS and OS was tested in univariate and multivariate analyses adjusted for clinicopathological parameters. Univariate model of DFS did not detect any association between Gli1 expression and clinical outcome (Table 3, Fig. 1E). On the other hand, using Kaplan–Meier analysis we found that patients with positive Gli1 expression had an unfavorable overall survival prognosis; the median overall survival in this population was of 35 months, while it remained undefined for negative–Gli1 patients, as more than 50% of them were still alive at the time of analysis (P = 0.04) (Table 4, Figure 1F). Notably, in the multivariate model, Gli1 nuclear expression retained an independent negative prognostic role for OS (p = 0.04, Table 4).

**Table 3:** Univariate and Multivariate analysis of factors affecting DFS in advanced serous ovarian cancer.

Variables	Univariate		Multivariate	
	HR (95%CI)	P	HR (95%CI)	*P
**Age** **(yrs)**	0.8 (0.4–1.5)	0.40	–	–
≤54				
>54				
**Tumor grade**	1.0 (0.5–2.2)	0.90	–	–
G1–2				
G3				
**Residual tumor**	2.9 (1.5–6.0)	**0.003**	2.6(1.4–5.0)	**0.003**
0 mm				
>0 mm				
**Chemosensitivity**	67.4 (21.4–212.1)	**<0.0001**	11.5(5.1–25.9)	**<0.0001**
Sensitive				
Resistant				
**Gli1 expression**	1.6 (0.8–3.3)	0.20	1.2(0.6–2.3)	0.6
Negative (≤10%)				
Positive (>10%)				

DFS = Disease-Free Survival. HR = Hazard ratio. CI = confidence interval. *Ps were derived from the COX proportional hazards model. Only variables with p-value ≤0.2 in the univariate analysis were included in multivariate model. χ^2^ of the model = 41; p value <0.0001.

**Table 4:** Univariate and Multivariate analysis of factors affecting OS in advanced serous ovarian cancer.

Variables	Univariate		Multivariate	
	HR (95%CI)	P	HR (95%CI)	*P
**Age (yrs)**	0.8 (0.3–1.8)	0.60	–	–
≤54				
>54				
**Tumor grade**	0.8 (0.3–2.0)	0.60	–	–
G1–2				
G3				
**Residual tumor**	1.8 (0.7–4.2)	0.19	1.4 (0.6–3.3)	0.4
0 mm				
>0 mm				
**Chemosensitivity**	7.5 (2.6–21.9)	**0.0002**	3.9 (1.7–9.3)	**0.002**
Sensitive				
Resistant				
**Gli1 expression**	2.7 (1.1–7.1)	**0.04**	2.2 (1.0–5.1)	**0.04**
Negative (≤10%)				
Positive (>10%)				

OS = Overall Survival. HR = Hazard ratio. CI = confidence interval. *Ps were derived from the COX proportional hazards model. Only variables with p-value ≤0.2 in the univariate analysis were included in multivariate model. χ^2^ of the model = 14.3; p value = 0.0025.

### Classical Prognostic Factors in Advanced Serous Ovarian Cancers

The prognostic role of age at diagnosis, histological grade, residual tumor, and chemosensitivity for both DFS and OS was tested in univariate and multivariate analyses (Tables 3 and 4). The presence of any residual tumor at primary surgery (>0 mm) [21] and platinum-resistance were found to be associated with a higher risk of recurrence of disease, in both univariate (p = 0.003 and p<0.0001, respectively), and multivariate analyses (p = 0.003 and p<0.0001, respectively). Platinum-resistance was also found to be an unfavorable independent prognostic variable for OS (p = 0.002).

### Gli1 is Expressed in Ovarian Carcinoma Cells

**Figure 2:**
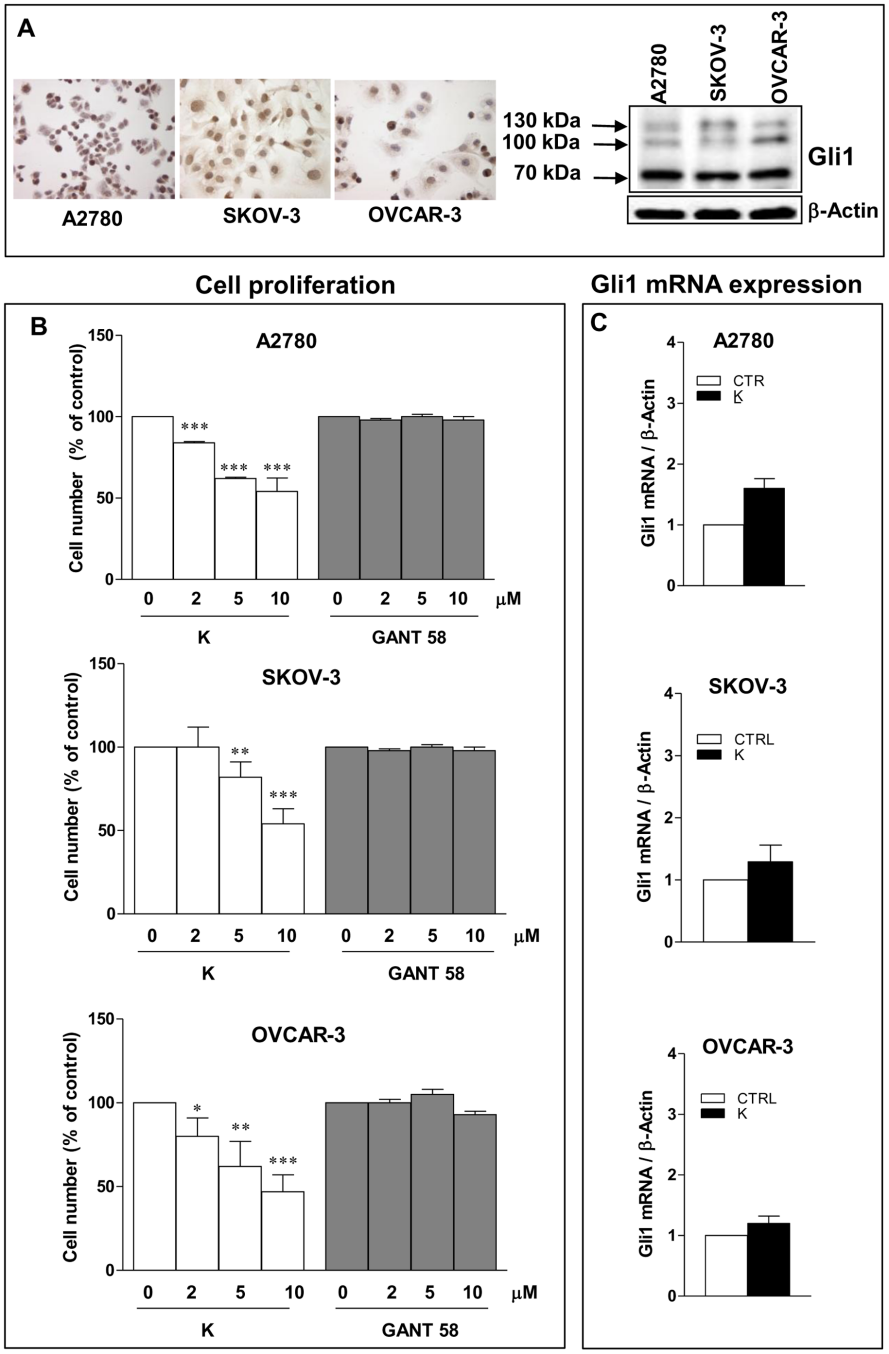
Effect of KAAD-cyclopamine and GANT58 on Hh pathway in human ovarian carcinoma cells. (**A**) Three human ovarian carcinoma cell lines, A2780, SKOV-3, and OVCAR-3 were subjected to immunostaining with antibody against Gli1. All of three cell lines express Gli1. Protein expression was also confirmed by western blot analysis. β-Actin was used as a loading control. (**B**) Cells were cultured in 2% FBS medium and treated with 2, 5 and 10 µM KAAD-cyclopamine (K) or GANT58 for 48 h. KAAD-cyclopamine treatment inhibits cell proliferation in all of three cell lines whereas GANT58 does not affect cell growth. Shown are cell number expressed as percentage of DMSO-treated cells (mean ± SD from three different experiments; *P<0.05, **P<0.01, ***P<0.001, t test). (C) Gli1 mRNA expression was evaluated by Q-PCR analysis in ovarian cancer cells treated with 10 µM KAAD-cyclopamine for 48 h. Data are expressed as fold change vs. each respective Control (CTRL), taken as calibrator for comparative quantitation analysis of mRNA levels. Each sample was measured in triplicate, the experiment was repeated 2 times with similar results. Graphics show mean ± SD.

To investigate a putative role for Gli1 in ovarian cancer, we first determined protein level in three human ovarian carcinoma cell lines A2780, SKOV-3 and OVCAR-3. Immunocytochemical analysis showed Gli1 nuclear staining in the three cell lines (Fig. 2A). Protein expression was next confirmed by western blot analysis using a commercial antibody raised against amino acids 781–1080 of Gli1 of human origin (Figure 2A). The antibody recognized diverse forms which were almost visible in all cell lines: the 130 KDa isoform likely acting as activator, the weak repressor 100 KDa [22], and an additional 70 KDa band not yet identified, but also reported in malignant pleural mesothelioma cell cultures [23].

### Treatment with KAAD-cyclopamine Inhibits Cell Proliferation of Ovarian Carcinomas Cells

In order to examine if the activated Hh pathway is essential for the proliferation of ovarian carcinoma cells, A2780, SKOV-3 and OVCAR-3 were exposed to increasing concentration of KAAD-cyclopamine, a compound able to inhibit both Hh ligand-dependent and independent pathway activation, through direct interaction with Smo. Direct counting of viable cells showed that KAAD-cyclopamine induced a dose-dependent reduction of ovarian tumor cell proliferation (Fig 2B). Assessment of cell viability by MTT assay confirmed the inhibitory effect of KAAD-cyclopamine on A2780 cell growth, whereas no inhibitory effect was observed for SKOV-3 and OVCAR-3 cells (Fig. S1). An over- or under-estimation of MTT results if compared to cell count results has been already reported by several Authors, the major contributory factors to this discrepancy being represented by differences in cell size, cell morphology, cell growth (cluster or monolayer) or mitochondrial activity [24]–[26].

### Effect of KAAD-cyclopamine on Hh Signaling

To confirm that the reduction of cell proliferation *in vitro* by KAAD-cyclopamine was due to specific inhibition of the Hh pathway, we analyzed the change in mRNA and protein Gli1 levels in drug-treated ovarian carcinoma cell lines. Q-PCR analysis showed that treatment of A2780, SKOV-3 and OVCAR-3 with 10 µM KAAD-cyclopamine did not significantly modulate Gli1 levels after 48 h of exposure (Fig. 2C). The lack of an effect of KAAD-cyclopamine on Hh signaling activity was additionally confirmed by immunoblotting (data not shown). These data suggest that the inhibition of ovarian cancer proliferation *in vitro* by KAAD-cyclopamine is not a Smo-mediated event.

### Treatment with GANT58 does not Inhibit Cell Proliferation of Ovarian Carcinomas Cells

In order to confirm the lack of effect of Hh pathway modulation on ovarian cancer cell proliferation, A2780, SKOV-3 and OVCAR-3 were exposed to increasing concentration of GANT58, a specific Gli1 inhibitor. Results obtained showed that GANT58 did not induce any reduction in ovarian tumor cell proliferation (Fig. 2B). These data were confirmed by MTT assay (Fig. S1).

## Discussion

Although the Hh pathway activation has been shown in various types of malignancies [5], there are only few reports showing it in human ovarian carcinoma specimens [27]–[30], with only two examining the prognostic role of Hh signal molecules in ovarian cancer. Besides, results from these two studies were conflicting: Chen and colleagues [27] found that among Shh, Dhh, Ptch, Smo and Gli1, Dhh was the only factor associated with survival outcome, while in the Liao’s study [29] examining the prognostic role of Shh, Ptch, Smo and Gli1, the latter was the only protein independently associated to patients’ survival. No data are available at the moment that could help explaining this discrepancy, although it might be due to the analysis of different population in terms of stage and/or tumor histotype. In the present study we investigated the prognostic role of Gli1 in a homogenous population of advanced serous ovarian cancers showing that patients whose tumors express nuclear Gli1 staining (>10%) experience a shorter OS compared to negative cases (≤10%). The independent role of Gli1 expression as marker of poor prognosis was sustained by multivariate analysis, after adjusting for residual tumor at surgery and chemosensitivity. Overall our findings not only strengthen the hypothesis that Gli1 may represent a new important prognostic marker in ovarian cancer, but also suggest that Gli1-expressing tumors poorly respond to second-line therapies, thus supporting potential therapeutic usefulness of combinations of Gli-targeted agent with standard treatments in ovarian cancer patients.

In this context, we were prompt to examine if the activated Hh pathway is essential for the proliferation of ovarian carcinoma cells. To this end we firstly assessed by immunocytochemical and immunoblot analysis the expression of Gli1 in different ovarian cancer cell lines, i.e. A2780, SKOV-3 and OVCAR-3, these studies revealing similar protein expression in all 3 cell lines examined. Since cyclopamine inhibits Hh signaling by binding to and preventing activation by Smo [31], we next assessed the contribution of autocrine signaling to tumor growth by studying the effect of this drug on *in vitro* cell proliferation. Notably we found that, although this plant alkaloid induced a dose-dependent inhibition of cell proliferation in the cell lines tested, however this effect did not correlate with an inhibition of Hh signaling, as judged by the lack of Gli1 modulation in treated cell cultures. These findings suggest that the inhibition of cell proliferation was not the result of canonical Smo-mediated Hh pathway inhibition, but rather a nonspecific or toxic effect. In line with these data, no differences on cell proliferation were observed when cell lines were treated with GANT58, a specific Gli1 inhibitor. Notably, the lack of response of the SKOV-3 and OVCAR-3 cell lines to Hh pathway inhibition is consistent with previous results by our and other groups, using cyclopamine or a specific Gli1 inhibitor [32], [33]. Moreover, Kudo *et al*. [34] also recently showed that treatment with anti-Gli1 shRNA did not affect Gli1 expression in A2780 cells. On the other hand, our *in vitro* data stand in contrast to the studies of Liao *et al*. [29] and Kandala and Srivastava [35] demonstrating that, after treatment with cyclopamine, Gli1 expression was decreased in SKOV-3 and OVCAR-3 or A2780 cells, respectively. The reasons for these conflicting results remain unclear, adding another layer of complexity to efforts designed to ascribe ovarian tumor growth to excessive Hh activity. Although they may be due to differences in experimental systems (e.g. cell lines are prone to genotypic and phenotypic drift during their continual culture) and/or assay methods, it is also conceivable that they might actually reflect heterogeneity of autocrine and paracrine signaling in ovarian cancer. In this context, it is worthy to note that, along with staining of epithelial cancer cells, we also observed sporadic nuclear expression of Gli1 in the surrounding stroma (data not shown), this finding suggesting a cellular interaction between stroma and epithelium, with possible occurrence of paracrine signaling. Unfortunately, owing to the limited stroma component usually associated with high-grade lesions, we were not able to determine the extent of Gli1 expression in tumor microenvironment, and more studies are needed to better clarify the role of paracrine Hh signaling in ovarian cancer.

Actually, similar discrepancies in experimental results have been also reported in other tumors, such as prostate, pancreas, and colon [36], [37]. Yauch and colleagues [38] made the interesting observation that Smo-antagonist-mediated growth inhibition of a large number of cancer cell lines of epithelial origin does not correlate with Hh target gene expression in these cells, suggesting that a) the observed *in vitro* growth repression might be due to off-target effects when these compounds are used at high concentrations, and b) there is a paracrine requirement for Hh ligand signaling in the tumorigenesis of Hh-expressing cancers, with important implications for the development of Hh pathway antagonists in cancer.

More consistent are experimental data on the role of Hh pathway in mediating chemoresistance in ovarian cancer, with different studies showing implication of the signaling in resistance to cisplatin, taxane and epothilones [27], [33], [39]. This hypothesis is in keeping with recent data suggesting that Hh signaling regulates cancer stem cells (CSC), commonly supposed to have enhanced tumorigenicity and resistance to chemotherapy in comparison with non-CSCs [6], [37]. Notably, however, we did not observe any differences in Gli1 expression between sensitive and resistant patients.

In conclusion, our study highlights that evaluation of Gli1 expression in advanced serous ovarian cancer may provide clinicians with important clues to prognosis, a finding needing confirmation in large-scale prospective clinical trials. Nevertheless, it is clear that several questions on the precise role of the Hh signaling pathway in human ovarian cancer remain unanswered, including the relative contributions of autocrine and paracrine hedgehog signaling to tumor growth and progression, and the role of the pathway in the regulation of CSCs. Importantly, a clinical trial using GDC-0049 (Vismodegib), a small-molecule inhibitor of Smo, recently carried out in patients with epithelial ovarian cancer in second or third remission, did not show any increase in progression-free survival for vismodegib maintenance versus placebo. Noteworthy, there was a lower than expected prevalence of Hedgehog ligand expression in archival tumor tissue, and thus a correlation between Hedgehog ligand expression and clinical benefit could not be determined [40]. Altogether, these findings further strength the idea that for accurately testing future tumor therapies involving Hh pathway inhibitors, efforts have to be made to make sure to select experimental models that best represent human tumor. Results from more comprehensive studies on ovarian cancer Hh signaling will allow scientist to predict the feasibility of clinical trials of Hh signaling inhibitors in ovarian cancer, by identification of an appropriate patient selection strategy.

## Supporting Information

Figure S1 **Cytotoxicity of KAAD-cyclopamine and GANT58 on human ovarian carcinoma cells.** Cells were cultured in 2% FBS medium and treated with 2, 5 and 10 µM KAAD-cyclopamine (K) or GANT58 for 48 h. Inhibitory effects of KAAD-cyclopamine are observed in A2780 only. GANT58 does not affect cell viability in all of three cell lines tested. Data are shown as mean ± SD from three different experiments (*P<0.05, **P<0.01, ***P<0.001, t test).
(TIF)
