# Supplementary figures and images for: Expression of the Glioma-Associated Oncogene Homolog 1 (Gli1) in Advanced Serous Ovarian Cancer Is Associated with Unfavorable Overall Survival
Source: PLoS One. 2013 Mar 28;8(3):e60145. doi: 10.1371/journal.pone.0060145 (PMC3610749; doi:10.1371/journal.pone.0060145)

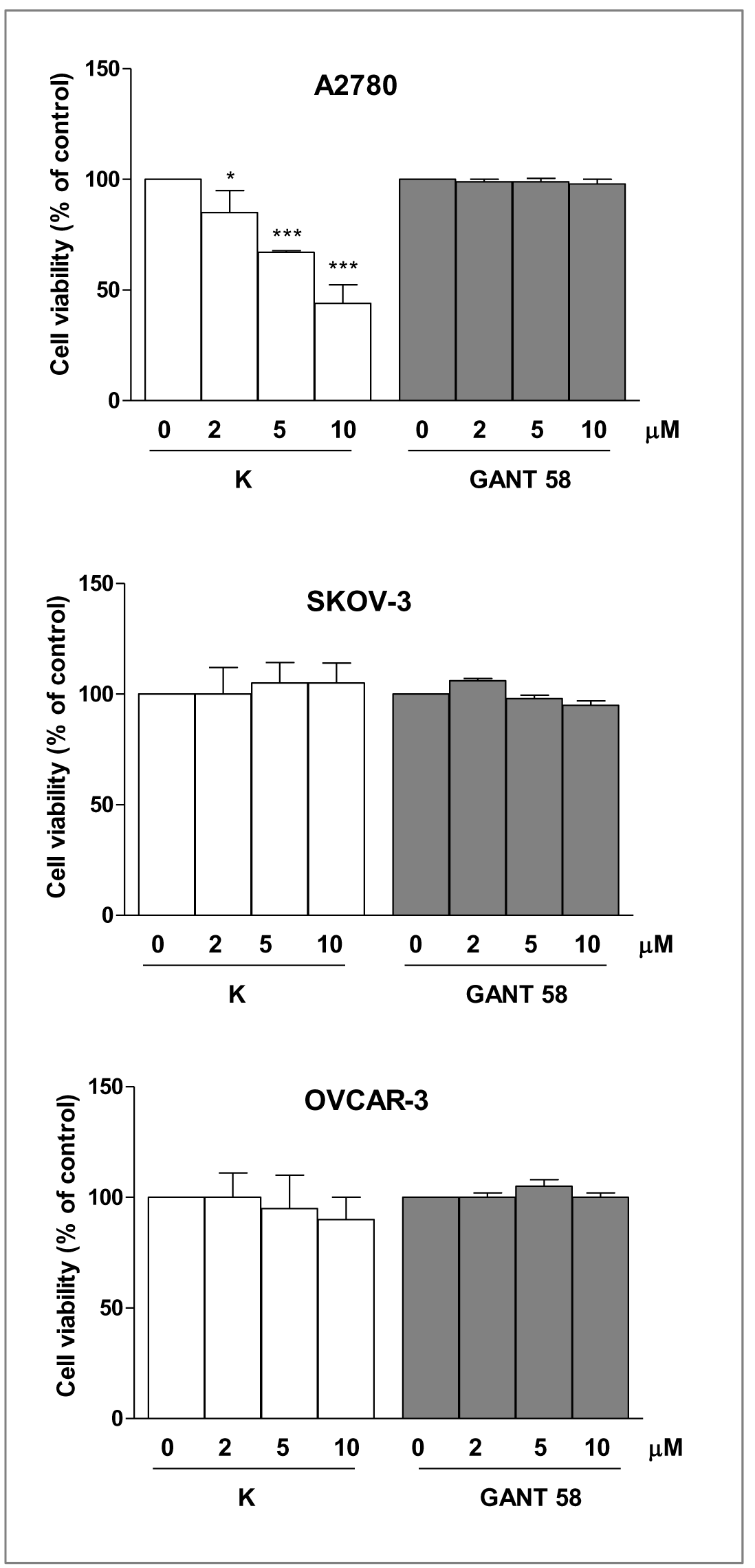

Supplement: Figure S1 — Cytotoxicity of KAAD-cyclopamine and GANT58 on human ovarian carcinoma cells. Cells were cultured in 2% FBS medium and treated with 2, 5 and 10 µM KAAD-cyclopamine (K) or GANT58 for 48 h. Inhibitory effects of KAAD-cyclopamine are observed in A2780 only. GANT58 does not affect cell viability in all of three cell lines tested. Data are shown as mean ± SD from three different experiments (*P<0.05, **P<0.01, ***P<0.001, t test). (TIF) [file pone.0060145.s001.tif]
